# “Oh, We Don't Want the Men Around.” The Experience of Men in Nursing During Prelicensure Labor and Delivery Clinical Rotation

**DOI:** 10.1155/nrp/5562479

**Published:** 2024-12-05

**Authors:** Kechi Iheduru-Anderson, Chimezie J. Agomoh

**Affiliations:** ^1^Division of Nursing, School of Rehabilitation and Medical Sciences, The Herbert H. and Grace A. Dow College of Health Professions, Central Michigan University, Mount Pleasant 48858, Michigan, USA; ^2^Department of Nursing, School of Nursing and Health Sciences, Curry College 1071 Blue Hill Ave, Milton 02186, Massachusetts, USA

**Keywords:** diversity and inclusion in nursing, inclusive nursing education, labor and delivery nursing, male nurses, men in nursing, nursing education

## Abstract

**Background:** The nursing profession has traditionally been dominated by females, with male nurses comprising a small minority. The labor and delivery unit is one area of nursing that is particularly associated with female care providers. Male nursing students face unique challenges and experiences while completing their clinical rotations in this setting. Understanding these challenges is vital to help support them during their clinical rotation. With the nursing shortage being a global concern, all students must be supported to achieve academic success, regardless of gender.

**Purpose:** This study uses social role theory to explore the experiences of male nursing students during their clinical rotations in the L&D unit.

**Method:** This study used a phenomenological qualitative approach to gather data through in-depth interviews with male nursing students who had completed their labor and delivery unit clinical rotations. The data were thematically analyzed.

**Findings:** The analysis identified five main themes: Culture, Exclusion from the Learning Experience, Different Expectations for Men in Labor and Delivery Units, Men are not Equipped to Deal with Emotions in Labor and Delivery Units, and (Mis)interpretation of Touch.

**Conclusion:** This study explored the experiences of male nursing students during their clinical rotations in the labor and delivery unit. The findings will add to the body of knowledge on gender and nursing education and provide valuable insight into the experiences of male nursing students, which can be used to support and improve nursing education in this area.

## 1. Introduction/Background

The nursing shortage is a global concern. Therefore, it is imperative to support all students, despite their gender identity, as they go through nursing education. Increasing the number of men entering and graduating from prelicensure nursing programs may help ease the workforce concern [1, 2]. However, despite the recent increase in men entering the nursing workforce in the past 3 decades, they remain significantly underrepresented. Globally, gender stereotypes discourage men from considering nursing as a viable career option, as they may feel that it does not align with the typical masculinity associated with men [3–7].

The nursing profession has traditionally been women-dominated, with men comprising a small minority (9.4%) of all United States (U.S.) nurses [8]. Labor and delivery (L&D) units are areas particularly associated with significant social role norm challenges, where students experience the most challenges while completing their clinical rotations during nursing education. This study explored men's experiences in nursing during their clinical rotations in the L&D units using social role theory. When referring to nurses, many invariably use gendered pronouns—her and she, unconsciously communicating that nurses should be women. *To avoid perpetuating these stereotypes, I have elected to use “men in nursing” and “men nurse(s)” as an all-inclusive language for students and those currently practicing.* The study aims to answer the research question: What is the experience of men in nursing during their rotation in L&D units in their prelicensure nursing education?

### 1.1. Research Question

What is the experience of male nursing students in L&D rotations?

1. The study aims to explore men's experiences in nursing during their prelicensure nursing program, focusing on how gender and gender norms and expectations affect their interactions with patients, colleagues, and other healthcare professionals in the settings.
2. How do men in nursing navigate their roles and responsibilities as students in L&D units during their clinical rotations?

## 2. Guiding Theory

### 2.1. Social Role Theory

Social role theory guided an in-depth review of retrieved articles and the development of an interview guide for this study. Social role theory, initially proposed by Eagly and Steffen [9], is a sociological and psychological theory that elucidates how social roles, norms, and expectations influence individual and group behaviors and attitudes. It suggests that gender differences in behavior and personality traits stem primarily from the distinct societal roles of men and women. Social role theory posits that people's behaviors are shaped by the stereotypical societal defined social roles they occupy based on characteristics such as gender, age, race, and social class. Specific expectations, obligations, and privileges dictate individuals' role in society. These expectations can shape how individuals think, feel, and behave in certain situations [10].

## 3. Literature Review

### 3.1. Cultural Expectations and Gender Norms

Traditional gender roles continue to influence the career choices of men and women. Men may feel societal pressure to pursue careers in more male-dominated fields, such as business, technology, or engineering. In contrast, women may feel more encouraged to pursue healthcare, education, or social work careers. In exploring the experiences of male nurses in the nursing profession, Kronsberg, Bouret, and Brett [10] and Evans [11] argued that men's experiences are influenced and challenged by prevailing societal definitions of masculinity, which act as barriers to entry and retention in the profession with dire consequences. Gender stereotypes can lead to a sexualization of touch from men in nursing, which can create a problematic situation for male nurses who are providing care to female patients. Male nurses may be perceived as a threat to female patients' privacy and dignity, or their touch may be viewed as inappropriate or sexualized [12, 13]. These led to increased anxiety, excessive caution, and frustration that may impact patient care [14]. Although touch from women nurses is generally accepted as part of nursing care, the sexualization of touch from men in nursing can create a problematic situation for male nurses who are providing care to female patients [1]. The deconstruction of gender stereotypes and sexualization of men's touch in intimate care moments is critical to nursing education and practice.

### 3.2. Male Masculinity

Masculinity is often associated with independence, strength, and competitiveness, which may not necessarily align with the traditional expectations of caring professions such as nursing, teaching, and social work. Men pursuing nursing may be seen as breaking conventional gender norms by embracing a caring and nurturing role reserved for women [15, 16]. Historically, society views traditionally female-dominated jobs as lower status, discouraging men from pursuing these nontraditional male careers [17–19]. As a result, men pursuing careers in these fields may face unique challenges related to their gender and masculinity [7, 12, 17, 20]. Studies have shown that men in nursing may encounter bias from patients, colleagues, or supervisors with traditional views of gender roles [4, 7, 21]. Gender bias and discrimination impede recruitment and retention of men into the profession [11, 12].

### 3.3. Attrition Rates of Men Nursing Students

While data on attrition rates among male nursing students are limited, research suggests that male students are more likely to drop out of nursing programs than female students [22]. Stott [23] suggests that male nursing students may face social isolation or discrimination within nursing programs, which can also contribute to higher attrition rates. Male students may feel they do not fit in with the predominantly female student body or faculty or may experience bias or discrimination from peers or instructors [10, 19, 24, 25].

### 3.4. Images Representing Nursing in the Media

Over the past decade, images representing nursing have become more diverse. However, the media often portrays them as subservient to doctors and other healthcare professionals. Although the COVID-19 pandemic highlighted the role of nurses in healthcare to the public, they are still shown as following orders and performing tasks that are delegated or assigned to them rather than taking a more active role in patient care. Even some nurses and nursing students believe their role is to assist doctors [17, 26]. The media representation of nursing can impact recruiting men to the profession. Traditionally, nursing has been seen as a female-dominated profession, and this stereotype has been perpetuated in the media [20, 24, 27]. Nursing has often been depicted as nurturing, compassionate work better suited for women, while men have been portrayed as more suited for careers in medicine or surgery. The sexuality of male nurses has been questioned. Some have been called “murse” [21]. Sasa [16] cautions that gender-based labels such as male nurses are injurious to the nursing profession, as they perpetuate gender stereotypes and reinforce the idea that nursing is a woman's profession, which can affect everyone in the profession.

### 3.5. Men in Maternal Childcare and L&D Nursing

Gender stereotypes are evident in the disproportionate representation of men in psychiatric and mental health nursing, emergency and trauma care, and, more recently, with their rapid ascent to “masculine-congruent leadership” positions compared to their female counterparts ([11], p. 321). Studies show that although men in nursing encounter various challenges during their prelicensure nursing education, L&D unit clinical experiences pose significant challenges [25, 28]. Men in nursing are barred from practicing in obstetrics and gynecology due to gender bias [28, 29]. Many nursing students viewed L&D nursing as a women's specialty [22]. Exploring the maternity clinical experience of 60 male Egyptian nursing students, the authors found that the participants preferred caring for male patients and found the experience of maternity nursing stressful due to the unfavorable attitude of the clinical instructors and the refusal of women to receive care by a male student [30]. The male nursing students reported feeling uncomfortable in performing the abdominal examination, breast examination, and perineal care. They found newborn assessment, attending cesarean section, and providing prenatal and postnatal education classes for mothers most interesting [30]. In a cross-sectional survey of 93 male student nurses from a Kuwaiti nursing college, 82.8% of the students reported being treated differently due to their gender. The intimate nature of care in the L&D unit is a concern for many male nurses and students [13, 31, 32]. Therefore, men prefer nursing specialties requiring less intimate patient touching. Unlike men in medical obstetrics and gynecology, men in nursing are subject to gender stereotypes and biases that view them as less competent or less caring than their female counterparts. Following social role theory thinking, one would argue that the more men in nursing, the more the profession will be viewed as gender-neutral.

There are many studies on men in nursing. Limited studies specifically explored men's experiences during the prelicensure clinical rotation in maternity, L&D. The current study aims to fill this gap in the literature by contributing to a more in-depth understanding of men's experiences in specific areas of nursing during their prelicensure nursing programs. The findings will be helpful in the development of strategies to support men in prelicensure nursing programs in this area and provide guidance to educators and unit managers aiming to improve the overall nursing education experience for these students. The study will also add to the knowledge of gender and nursing education. Findings may also help hospitals and nursing education programs to develop antidiscrimination gender policies to enhance the education of future nurses.

## 4. Methodology

### 4.1. Research Design

This study used a descriptive phenomenological qualitative approach to gather data through individual interviews with male nurses or nursing students who have completed their clinical rotations in the L&D units. Descriptive phenomenology is appropriate for this study as it aims to present the experience as intuitively and faithfully as possible, without presuppositions, allowing others to understand and empathize with it [33–35].

### 4.2. Ethical Consideration

The researchers obtained permission for the study from the Central Michigan University Institutional Review Board (IRB Approval #: 2023-386). Participants were provided informed consent via email to review. The researchers answered all their questions before they agreed to participate in the study. Instead of obtaining written consent, their verbal consents were recorded at the beginning of the interview to improve confidentiality. Participants were also reminded that their participation is voluntary, and they may withdraw from the study at any time, even during the interview. The researcher assigned pseudonyms to participants and removed any identifying information from the transcripts.

### 4.3. Participants Recruitment

Participants were selected through purposive sampling through social media, direct email to peers and colleagues, and soliciting participation through nursing organizations. To participate in the study, the person must identify as a man, a current student who has completed their L&D experience, or a licensed registered nurse who graduated from prelicensure nursing in the last 6 years. Participants were eligible if they identified as a man, including transgender men and gender-diverse individuals who identify as men. We recognize that gender identity is a complex and individual experience, and our criteria were designed to be inclusive of all individuals who self-identify as men, regardless of their gender assigned at birth. The 6-year cutoff coincides with the 15th anniversary of the Johnson and Johnson campaign for nursing's future, which helped reshape the image of nursing in the public and provided recruitment resources to attract and support a more diverse population to the profession [36]. The focus was on recruiting individuals with diverse experiences who could provide meaningful insights about their educational experiences within this particular context and were willing to share with the researchers.

Sample size in qualitative research can be determined by multiple factors, including the depth of inquiry, the scope of the research question, and the practical constraints of the study (e.g., recruitment, resources, and time) [37, 38]. Despite casting a wide net, only 11 men who met the inclusion criteria agreed to participate in the study. Four men were rejected for the study because they have been nurses for more than 6 years at the time of the data collect. The student participants came from different institutions. None of the registered nurse participants graduated from the same institution. Of all the participants, only two came from a state in the Southeastern region of the United States. Three of the registered nurses work in a state in Northeastern United States. The sample size focuses on recruiting participants with rich experience to answer the research question [33, 37, 39]. The researchers completed the recruitment and data collection between June 2023 and September 2023.

In our study, we defined rich information as insights that could illuminate the personal, emotional, and educational aspects of men's experiences during L&D clinical rotation in prelicensure nursing education. Participants were selected based on their ability to reflect on these experiences in depth, as well as their varied demographic backgrounds and willingness to articulate their experiences and provide detailed responses to interview questions after reviewing the details of the study prior to consenting to participate. Their willingness to engage with the PI in an interview demonstrates meaningful engagement with the topic.

## 5. Data Collection

The researchers created a demographic survey tool to collect demographic information from the participants. The information collected includes age, student status, nursing status, and years of practice as a nurse. Data about racial and ethnic identification were not obtained because the focus of the study is not to differentiate the experience based on racial or ethnic classification. Data to answer the research question were collected using individual unstructured interviews. Unstructured interviews using one or two broad, open-ended questions with probes are appropriate to elicit an in-depth description of the experience [33, 34]. The researchers used the two broad questions (see Table 1) to elicit an in-depth description of their experiences, thoughts, and feelings during their clinical education in L&D units.

Follow-up questions were used to explore the participants' responses in more depth. Prompts such as “Tell me more,” “Would you provide more details,” “You said…,” “Would you elaborate?” “Let us go back to what you said about…,” “How did you address this?” Many more follow-up questions based on the participants' responses and comments were used to dig deeper to elicit more information about the participants' experiences.

Different qualitative research traditions have varying views on how many participants are needed [33, 37, 39]. For this study, our sample size was based on achieving meaningful depth and diversity of perspectives within the research context. While the idea of saturation was considered, it was not used as the primary justification for the sample size. Instead, we focused on the principle of informational adequacy, where the goal was to gather enough data to answer the research questions thoroughly without aiming for exhaustive or definitive saturation [40, 41]. This approach aligns with the study's exploratory nature and the qualitative paradigm we are working within, where the emphasis is on obtaining rich, in-depth narratives rather than achieving statistical or theoretical saturation [42].

## 6. Data Analysis

Data analysis employed inductive thematic analysis outlined by Braun and Clarke [43, 44]. This approach identifies themes from the data that are devoid of predetermined categories or theoretical frameworks [42–44]. Following the constructive paradigm, the analysis focuses on understanding participants' perspectives from their lived experiences and their interpretations of those experiences [45]. Inductive thematic analysis enables researchers to co-construct stories about everyday lived experiences with participants and communicate their assigned meaning to their audience [46]. The data analysis process is iterative and begins with familiarization with the data. The steps involved in the data analysis are represented in Table 2.

Thematic analysis offers flexibility in exploring diverse experiences and perspectives but requires rigorous application to ensure validity and reliability [47].

### 6.1. Trustworthiness

Reflexivity is crucial in thematic analysis, necessitating researchers to acknowledge their biases and positionality [47]. The researchers employed reflexivity by maintaining a reflective journal containing thoughtful recordings of personal thoughts and field notes that provided detailed descriptions of what happened in the field. Additionally, the researchers used peer debriefing and feedback from colleagues with diverse perspectives in nursing education and those outside nursing to enhance the trustworthiness of the findings. During the interviews with the students, the researchers made a concerted effort to create a safe and nonjudgmental environment for the participants to share their experiences as authentically as possible, emphasizing the importance of their voices and experiences.

## 7. Findings

**Figure 1:**
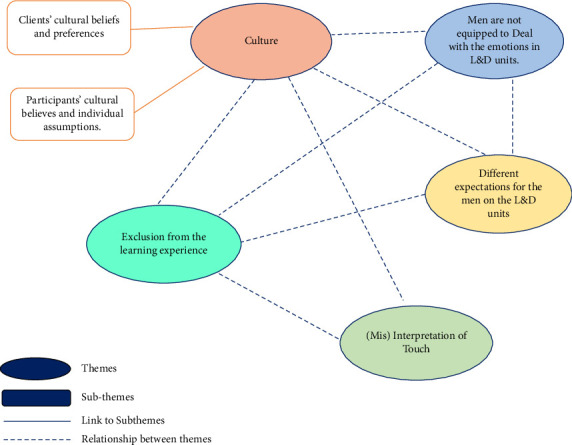
Thematic map of the findings.

Eleven participants were interviewed for the study. Due to the low number of participants, personal characteristics that may make the individual identifiable, such as age, racial identification, and student or nurse status, will not be included. There were four current nursing students and seven registered nurses. None of the registered nurses currently work in L&D or have ever been employed to work on the L&D units. Their age ranged from 23 to 36 years old. In the participants' quotes, “*male*” was replaced with “men” to be consistent throughout the paper. Five main themes and two subthemes were identified, as depicted in Figure 1. A detailed discussion about the themes and the supporting participant's quotes follows the discussion of the thematic connections.

### 7.1. Thematic Connections

The interconnected nature of the themes identified in this study reflects the complex experiences of men in nursing during their prelicensure L&D clinical rotations. The themes are not independent but deeply interrelated, with each theme both influencing and being influenced by the others.

### 7.2. Central Role of Culture

In this study, the theme of culture is foundational in shaping the experiences of men in nursing, particularly in L&D units during their clinical rotations. Nursing is a historically women-dominated profession, which creates inherent challenges for men entering the field. Cultural norms and societal expectations play a significant role in how men are perceived and treated in these spaces, often leading to their exclusion from learning opportunities. For example, societal gender roles tend to associate nurturing and caregiving with women, which can result in men being sidelined or discouraged from participating in certain aspects of the L&D process. This cultural backdrop directly contributes to the exclusion men experience in clinical settings, as both patients and healthcare professionals may expect men to refrain from engaging fully in care and support tasks in L&D units. Furthermore, these cultural and expectation-based contexts create additional challenges in how men's physical interactions are perceived. The interpretation of touch becomes fraught with potential misunderstandings stemming from gender norms and patient/institutional expectations regarding appropriate physical interactions.

### 7.3. Exclusion From the Learning Experience

Exclusion from the learning experience is directly connected to all other themes, highlighting its pervasive impact on men's experiences during clinical rotations. This exclusion manifests in various ways:

• Culture: Cultural expectations and gender norms in L&D units often exclude men students from certain learning opportunities.
• Different Expectations for Men in L&D Units: These differing expectations, rooted in cultural assumptions, contribute to men's exclusion from specific tasks or patient interactions.
• Men Not Equipped to Deal with Emotions: Men's exclusion from certain learning experiences can affect their emotional preparedness for working in emotionally intense environments like L&D units. Exclusion from direct involvement in patient care or emotional support situations may prevent men from developing the emotional intelligence and coping mechanisms needed in such high-stress and emotionally charged environments. This lack of exposure to emotionally intense aspects of the nursing role leaves many men feeling unprepared to manage the emotional demands of the job, such as offering empathetic support to patients during labor. The perception that men are less capable of handling emotional situations in L&D units contributes to their exclusion from emotionally charged scenarios, limiting their overall learning experiences.
• (Mis)interpretation of Touch: Concerns about how men's touch might be perceived can lead to their exclusion from hands-on learning opportunities, especially in intimate care situations.

### 7.4. Different Expectations for Men in the L&D Unit and Men Not Equipped to Deal With Emotions in L&D Units

The themes of *Different Expectations for Men in the L&D Unit* and *Men Not Equipped to Deal with Emotions in L&D Units* are directly connected, as these themes often reinforce each other. Men in nursing face distinct expectations that differ from those placed on their female counterparts. Research suggests that women nursing students frequently demonstrate more emotional depth and reflective learning [48], which can marginalize men who may not align with these expected emotional responses. These differential expectations contribute to the perception that men are unprepared to manage the emotional complexities of L&D settings. The belief that men are less emotionally equipped for L&D work leads to different treatment and responsibilities, which, in turn, perpetuates the notion that men are not suited for the emotional aspects of care in the L&D environment.

### 7.5. (Mis)interpretation of Touch

Physical touch is an integral part of patient care in L&D. However, due to gendered expectations regarding appropriate physical boundaries and roles, any physical touch by male nurses may be more heavily scrutinized or misinterpreted compared to their female peers. The stereotype that men should not be as emotionally or physically involved in caregiving roles can lead to situations where their touch is perceived as inappropriate or uncomfortable, even when it is entirely professional and part of nursing care. This misinterpretation of touch further exacerbates the challenges men face in navigating their roles within the L&D setting.

### 7.6. Culture

The first theme, “Culture,” refers to how clients' and participants' cultural beliefs, assumptions, and preferences influenced participants' experiences in the L&D units. Culture was crucial in how the participants experienced their L&D clinical rotation during their prelicensure nursing education. Two subthemes, “Clients' cultural beliefs and preferences” and “Participants' cultural beliefs, individual assumptions, and attitudes,” are used to describe them in detail.

#### 7.6.1. Clients' Cultural Beliefs and Preferences

Respecting clients' cultural beliefs and preferences is critical for patient-centered care. All the participants reported that the client's cultural beliefs, practices, and preferences affected their learning in the L&D clinical rotation. The participants described specific patient preferences for

Female caregivers during their stay in the L&D units are due to cultural or personal beliefs. For example, the two participants described specific incidents of patient and family preference that prevented them from participating in the clinical experiences. *For P-01*,

When a patient said they don't want a man in the room, you must respect that. I understand that. In this case, you have to go with what the patient and family want.

P-10 stated that while some of the birthing parents have no problem with a man providing care, some of their partners were against it. They stated:

“In this case, if the partner says no, you have to leave. Some patients don't care; they just want to have healthy babies, good outcomes, and someone who is professional and caring.”

Almost all the participants said they respected the patient's wishes not to be cared for by men on this unit.

#### 7.6.2. Participants' Cultural Beliefs, Assumptions, and Attitudes Toward L&D Experiences

Participant's attitudes, cultural assumptions, and perceptions of the role of men in nursing, specifically in L&D units, contributed to the experience. Three participants expressed no desire whatsoever for the L&D experience and felt that “men have no business in this unit.” P-11 stated:

“I could have done without that part of nursing school. I was never going to work in L&D, so it made no difference. I don't even think I got a single question on maternity in my NCLEX.”

A similar sentiment was expressed by P-06 about their experience. They said:

“Men have no business in the L&D. What type of man wants to work there all the time? You know what I mean? I wouldn't want some pervert watching my wife's intimate moment or examining her.”

This participant was asked how they felt about physicians examining a birthing person in L&D. They stated, “That's different. The doctor has more education and has spent more time with the patient and maybe the OBGYN.” However, three participants did not want any part of the L&D experience from a socially gendered norm perspective. P-10 explained that in their culture,

It's not men's business to be near while the women are giving birth. For clinical, I specifically told my instructor that I didn't want to go to it, but she said I still needed to go because it was required. I witnessed one C-section, and that was enough for me.

P-03 had a different take on the role of men in L&D. They stated:

“I think that this is the area (L&D) that should be left for women. It will make them feel that this is their area—something they can call their own.”

A similar sentiment was shared by P-02, but they extended the thought, noting that

Other women who have been through the birthing experience would better understand the emotions of other women in the same situation and are better suited for L&D work. I will never give birth… so, I don't think it my place. I don't think it's the place of men to take that over from women; I think it should be left to the women.

Participant P-02 further clarified the above statement, saying

“Representation matters; professionally, men can take care of women, but they will never understand the process or what the woman is going through. So, it is about representation.”

### 7.7. Exclusion From the Learning Experience

This second theme exclusion from the learning experience described participants' experiences in the L&D units. Most participants told stories of exclusion from the learning experience due to their gender. Four participants were excluded from observing a live virginal birth or performing abdominal or virginal examination because the nurses on the unit thought it was not appropriate for them to see the patient in such a vulnerable position. P-11, who did not wish for the L&D experience, reported being excluded from actively engaging in most of the experience as their women peers. They stated:

I was excluded from observing examinations and life births. I was okay with it. While other students were getting the life experience, I read about it in the book and practiced exam questions. I had no interest in specializing in mother or baby care, so I did not care that I was missing out.

They believed that clinical instructors and nursing staff in the unit tended to project their personal bias and preference when deciding whether to allow men to participate fully in the learning process. As exemplified by P-01's statement, “They don't even ask the patient. My instructor told me, you are a big guy, and the patients may not want you in the room.” Some participants experienced persistent exclusion from participating by the patients, family, instructors, or unit nurses. P-07 explained

So, as a man, I was only able to experience a certain amount because the father and the mother usually be like, “Oh, we don't want the guy around,” because of how uncomfortable they were after the birth, how they looked, how they felt, their beliefs. So, it was a little troublesome. But all in all, I did get to experience some.

Some of the participants compared their inclusion and exclusion experiences in L&D to their experiences in other nursing units during their training. They felt that the nurses and the patients did not want men around the unit here because so much intimate caring was happening.

Whereas in med-surg and ICU, I was deeply involved in the care. However, as a student (in the L&D unit), it was hard to put the information to practice. So, as a man with very little opportunity for hands-on, it was a challenging learning experience. [P-09]

P-06 reported about their learning experience during the L&D,

“The way some of the nurses treat you or scrutinize you, you feel dirty. Like you have done something wrong. In that type of situation, I prefer not to be present because I need to protect myself.”

P-06 elaborated more about the exclusion from the learning experience. They explained that some men may not want another man to see their wives in such vulnerable situations.

They were uncomfortable with it. So, the stigma set around men in OBGYN is often based on our sexuality and how different we are as men and women. So, they're like, “Oh, I don't want you to see my wife. I don't want you seeing my partner in this condition,” or “I don't want you seeing me that way.” “So, I respected that. Moreover, I don't want to be accused of something nefarious.”

The language used when introducing the students to the patients and families could convey a message of exclusion. For example, P-08 said:

“No one says to the patient, “I have a female student working with me today.” But they will say, I have a male student working with me.” The fact that the instructor or the preceptor needs to qualify my presence with “I have a male student” is a problem. It sends a message that something unusual is happening.

The participant further explained.

When the instructor feels the need to qualify the statement with gender or sex, then that is a problem. It is like going to a patient and saying, I have a Black or white student nurse working with me. Would it be okay if they come in and learn?

Although many participants discussed being excluded from the experience, three had different experiences. Their instructors and nurses on the unit were welcoming and provided various learning opportunities for the students. The students described the experience as a

“very good experience. Everyone was helpful. The nurses on the unit invited me to observe while they examined the patients. I was allowed to help bath and weigh the baby; that was cool.”

Participant P-05 recounted an experience with a Spanish-speaking patient and an opportunity to practice therapeutic communication, bedside manner, and patient-centered care, which they remarked was essential for building trust with the patients. Men have something to contribute to L&D. Individual biases should not be projected onto the patients.

The participants described their exclusion experiences as mostly hands-off. As P-01 explained, “The whole semester felt pretty hands-off. We were mainly observing L&D.” The student described the entire maternal and child experience as “somewhat fun and boring.” P-04 stated, “I felt robbed of valuable learning experience. I deserved the same learning experience as my peers. But I got none of that.” P-09 attempted to explain the reason for the mostly hands-off experience. They described the L&D units as high-stress units where the nurses do not trust the students with a basic assessment. They explained that the liability may be too high for the nurses to allow the students to participate fully. Several men reported being observers compared to their women peers.

### 7.8. Different Expectations for the Men in the L&D Units

The participants described feeling that their instructors and unit nurses had different expectations of them. The expectations were not for them to perform better but for them not to be interested in the clinical experience, as P-01 explained

It was interesting, and I felt, as a man and a nursing student, I felt more singled out. There was a feeling or expectation that I wouldn't want to have anything to do with L&D from some of the nurses I followed. They just expected me to stand in the corner of the room and not do anything or say anything, which I kind of understand. They were really a tight-knit group there.

P-08 added about the different expectations,

I didn't feel like it was malicious on their part, but there was an assumption that I didn't want to partake in the care and that there was something wrong about me wanting to in some way. I got a little talking to by my instructor because I kept asking to do an assessment or even place the fetal heart monitor on the patient, but the nurses refused.

However, three participants said they did not mind being excluded from participating in the care. P-06 said, “They did not want me to do anything, touch the patient, or be in the room; I am good with it. It gave me more time to study, especially if an exam is coming up.” The staff and instructor's perceptions influenced access to patients.

From all the narratives, the participants related starkly different experiences between their experience and those of the physicians who were present and those they encountered during their clinical experiences. Many attributed different experiences to historical gender stereotypes, societal norms, and the composition of the professions. Gender bias in nursing is more evident in the L&D units where all the patients are women. According to P-10,

“There's the expectation that men are the nurses for when people start to get violent or heavy patients need lifting, the size and strength difference assumptions that are certainly not always true. Men enjoy tender moments, too.”

Some of the participants encountered assumptions about their competence and motivations for being in the L&D unit. P-08 discussed the irony of not being allowed to perform physical assessment by pointing out. “I also found it funny that many of their doctors were male, and all the nurses were female. These doctors were doing assessment, and no one was concerned about impropriety.” The student went on to elaborate.

Society accepts when the doctor is a man, so no one finds it unusual if they come to the L&D unit. But when a nurse is a man, and they are in the maternity unit, there is a lot of brow raising. If you want to specialize in this area, there are unkind questions about your sexuality. [P-08]

P-09 added that nursing is the one profession where people make fun of you and question your manliness in all healthcare. If you say you want to be a midwife, they snicker. But

P-01 described the only man they saw working once on the unit as “A very high energy, flamboyant personality guy; I could see how his attitude made it easier for him to integrate with the people there.” The participant continued, “He could have been a ped's nurse and worked with kids because he just had this bright effect that worked really well for that.” The nurse's personality was effeminate, making them acceptable to the nurses on the unit. In other words, men in nursing who exhibit some feminine attributes will be suited for L&D. Even among men in nursing, some believe that some characteristics are better suited for men who want to work in L&D or with children. This double standard about the role of men in nursing and students in L&D was not only from staff, but two participants expressed concerns about any man in nursing who wants to work full-time in L&D.

### 7.9. Men Are Not Equipped to Deal With the Emotions in L&D Units

Almost all the participants but one mentioned the emotions and feelings of the birthing parents and their partners. Some mentioned that men may be poorly prepared to handle “intense emotions present in the maternity unit.” P-08 explained, “Maybe they think men are not equipped to deal with the unique emotional challenges inherent in L&D units.” P-03 stated about the place of men nurses in L&D units.

Working in L&D can be emotionally intense during the delivery process, as well as supporting the mother and family members. Men in nursing may not be fully equipped to face these unique and intense emotionally charged situations. It takes a certain type of man to develop strategies for managing such emotional situations in a gender-sensitive manner. [P-03]

All participants expressed an understanding that their gender does not determine their ability to provide safe, competent, and compassionate care to parents during childbirth, after birth, or at other times. They noted that men show care and nurturing differently and that their way of caring may be misunderstood or viewed as below the level of care provided by women in similar situations. They noted that men's use of humor in these stressful situations may be misunderstood as tasteless and inappropriate.

### 7.10. (Mis)interpretation of Touch

Touch is inevitable in nursing, and therapeutic touch can be comforting to many patients in various circumstances. However, in some situations, touch could be misinterpreted. Four participants expressed concerns about being involved in intimate care situations such as childbirth, palpations, and vaginal examination. A few participants stated that although making sure that a woman is not bleeding excessively and fundal massage may be necessary, they do not feel comfortable participating. Some participants felt that the patients would not want a man to perform such activities either.

“While they may be okay with me observing the childbirth with so many people in attendance, I do not think that they will be comfortable with me performing such intimate examination.”

Said P-07. P-02 added to the concern about touch.

“You know, as a man, you want to be careful. You can be accused of something you did not do. I will never go into any room or do anything without my instructor in the room.”

Although several participants raised concerns about patients, families, and nurses, two participants thought that men should not want to engage in such intimate care activities. P-06 stated that men interested in working in L&D should be thoroughly vetted to establish their motives. When asked to elaborate, they said

There are several areas of nursing where a man can work without any issues or constant intimate contact with females. Why would a man want to work in this specialty all the time? That is just weird. (laughs). I would not want to see my woman, my wife, in that situation if they are giving birth. I also do not want to be doing that. [P-06]

This participant explained that the nurses on the maternity unit feel liable for the students' actions and, therefore, may want to have an extra layer of protection for their patients when there is a man nursing student on the unit. They explained, “Because just the male/female interaction seems more dangerous, maybe they think a man might try and do something wrong.” P-03 strenuously explained how vital the role of clinical instructors is in protecting the students from being accused of inappropriate behavior during the L&D clinical experience.

“The instructors need to protect the male students from being accused of inappropriate touch. She has to ensure that the students work within the established boundaries of therapeutic touch.”

One student was concerned with the level of close monitoring they experienced during clinical, especially during the maternity rotation. The student stated about this experience.

Every time I turned around, the instructor or nurse was there. Even after we had been checked for the procedures, they still monitored me very closely, as if I could not be trusted to be alone with female patients. It was a little creepy, and it made me extremely nervous. [P-03].

Contrary to the perception about the misinterpretation of touch in the L&D, one participant explained that they are not concerned about that.

### 7.11. Summary

Participants in this study provided in-depth description of their experiences during their rotation in L&D units during their prelicensure nursing education. Five key themes identified from their experiences: Culture, Exclusion from the Learning Experience, Different Expectations for Men in the L&D units, Men not Equipped to Deal With Emotions in L&D Units, and (Mis)interpretation of Touch. The implications of these findings are discussed in the following section.

## 8. Discussion

The purpose of this study was to explore the experiences of male nursing students during their clinical rotations in the L&D unit using social role theory. This section will discuss the major themes and their respective subthemes of this study in the current literature. Study limitations and recommendations for future research will also be addressed. Finally, implications for nursing practice, education, research, and health policy will be presented.

### 8.1. Summary of the Findings With Comparison to the Previous Literature

The cultural beliefs, preferences, and attitudes of clients and participants in the L&D units play a crucial role in participants' experiences. Participants in this study described how clients' cultural beliefs, practices, and preferences affected their learning experiences in the L&D units. A new finding in this study is some participants believe that there is no place for men in L&D for various cultural and personal reasons. Some participants, despite being nurses or nursing students, still subscribed to the stereotypical and biased perception that men who express interest in working in L&D units are deviating from traditional gender roles.

Participant's attitudes, cultural assumptions, and worldviews about the role of men in L&D units contributed to these beliefs. Their attitudes were influenced by their role in socialization and personal assumptions about the place of men in nursing. Their socialized roles, cultural upbringing, beliefs, and customs contributed to how they experienced their L&D clinical rotation.

Social role theory guided the current study. Social role theory explains that when labor is divided based on gender, behavioral distinctions arising from these roles are frequently incorrectly attributed to inherent gender differences [9]. Attributing these social group variances to biology rather than social factors deepens stereotyping and prejudice [49]. Many participants used the “she” pronoun when referring to the clinical nursing instructors and conveying the socially gendered role of expecting clinical nursing instructors to be women and L&D instructors to be women. Although the study had a small number of participants and not all who participated agreed with the gender norm, more than half of the participants expressed a typical sentiment about the role of men in nursing, especially in L&D.

The participants in this study felt that introducing them as male nurses in the clinical setting indicated that they were an anomaly that did not quite belong. A similar finding was reported by Whitford et al. [7] whose participants felt the use of the term “male nurse” to be irregular, reinforcing the gender stereotype about the profession. Studies have shown that men in female-dominated professions, such as nursing, may encounter bias from patients, colleagues, or supervisors with traditional views of gender roles [4, 7, 21]. These findings are congruent with the findings in this study.

Participants in this study reported exclusion from the learning experience due to their gender. They also reported different expectations between them and their female counterparts during their clinical experience in the L&D units. Some participants felt the instructors and nurses projected their preferences onto the patients. Even those who reported good experiences told stories of differential treatment and expectations. The exclusion reported by some of the students in this study has been previously reported in the literature [28, 30, 50–52]. The students in Cudé [28] reported that the clinical instructors did not support them in the labor, delivery, and newborn units. Although excluding the student from observing childbirth and hands-on experience of assessing and caring for the parent after childbirth was framed as protecting the students, Eswi and El Sayed [30] participants viewed it as clinical nurse educators not being supportive of student nurses in this area of nursing, therefore excluding them.

In this study, the participants reported being constantly watched, not being allowed to be in the room with female patients, or being suspected of nefarious deeds. The negative portrayal of men in nursing in the media as homicidal, corrupt, or incompetent and questioning of their masculinity and sexuality may contribute to the hyperscrutiny experienced by the men [20, 53].

The patient challenged the masculinity of the participants, viewing some of them as effeminate for pursuing a career in nursing and expressing interest in L&D. Even one of the participants reported that the only man he saw working in the L&D unit had “flamboyant personality” and they could see how he could easily integrate on the unit than the more masculine, less effeminate men. Their sexuality was also questioned. The same sentiment was reported by participants in previous studies [54], where a participant was asked if they were gay by a patient because it was strange for a female to be a nurse.

Most of the participants expressed concerns about being involved in intimate care situations that involve touching the patients. They felt uncomfortable engaging in such intimate care activities. This was based on their belief that patients would not want a man to perform such activities and their personal belief that men should not engage in such intimate care activities.

Furthermore, they reported that their clinical instructors closely monitor them to protect them from being accused of inappropriate behavior during the L&D clinical experience. These findings were similar to reports from literature where male nurses' touch was viewed as inappropriate or misinterpreted as sexual due to the stereotypical assumptions about men [12, 13]. The sexualization of touch from men in nursing can create a problematic situation for male nurses who are providing care to female patients [1].

Although studies on the experience of men in nursing during their L&D clinical experiences are limited, a troubling but significant finding is one participant's insistence that a man interested in working in the L&D unit or with a newborn has ulterior motives. The belief that interests in L&D by a man is perverted. This expression was reported in media portrayals of men in nursing as socially or sexually deviant [16, 20, 55].

Overall, the participants explained that the experience is highly contextual, depending on the type of setting, the instructor, and the staff nurses on the units. How the instructors approach the patients could influence how they respond to men nursing students assisting in their care. The work environment and type of setting contributed significantly to how the students experienced their L&D clinical. Those who worked in academic medical centers had a more supportive team and workplace culture that welcomed men nursing students than those who went to nonacademic medical centers or community hospitals. Even when clinical instructors and patients were willing to let the students into the delivery suite, some staff restricted students' access to the experience.

According to social role theory, the slow increase in the number of men entering nursing despite great promises for job security and diverse specialty options is related to societal views on gender roles. Toxic masculine stereotypes affecting the recruitment and retention of men in nursing and specialties like L&D include medical school rejects, assumptions of being gay, emasculated, and heterosexual deviant [16] (p. 595). Therefore, many young men may feel that the nursing profession does not align with traditional masculine roles, heightening the low sense of belonging they may experience in a female-dominated discipline. Social role theory suggests that increasing men's representation in nursing can positively impact the profession and help break traditional gender stereotypes.

### 8.2. Implications of the Study for Nursing Education and Practice

The statement from one of the participants about managing patients' emotional needs indicates a need for more education and practice on managing emotions and supporting patients in their needs. Emotional challenges are part of nursing, regardless of the specific unit or specialty. Both men and women nurses are trained to assess patients' needs and provide compassionate care and support to patients and their families in various settings and situations, including L&D units. Nurses, regardless of gender, are trained to provide emotional support, comfort, and care during L&D, as well as in other sensitive situations in healthcare. Each patient has unique emotional needs during L&D, and nurses, regardless of gender, must adapt care accordingly through active listening, assessing emotional well-being, and providing appropriate support. Understanding the root of such beliefs is important for nurse educators, managers, and leaders.

Nursing must continue to promote diversity and inclusion, recognizing that nurses, regardless of gender, bring unique skills and perspectives to patient care. Patients' preferences for providers may vary, and some may feel more comfortable with a nurse of a specific gender. When feasible, respect for patients' preferences is essential for evidence-based practice. However, ensuring that all student providers, including men, are adequately trained and competent to provide exceptional care in L&D units is also essential.

Men in nursing can and do provide empathetic and individualized care by creating a safe and supportive environment for patients. Effective communication and empathy are crucial skills for nurses working in various settings. These skills are not gender-specific but are developed through education, experience, and ongoing professional development. Men in nursing can fully develop and utilize these skills to provide emotional support to patients and their families. The L&D units are interdisciplinary environments where nurses work closely with other healthcare professionals. The emotional challenges are addressed through collaborative efforts of team members' expertise. Gender does not limit nurses' ability to collaborate effectively and provide holistic care.

While some patients may have personal and cultural preferences for a particular gender of healthcare provider during L&D, many others prioritize professionalism, expertise, and compassionate care over the nurse's gender. Nursing instructors and staff nurses on these units must set aside their personal biases and facilitate learning opportunities for all students. Nursing programs may use the findings to develop more inclusive and supportive learning environments that address the specific needs of men in nursing during clinical experiences. Clinical instructors can use targeted interventions and support to help men navigate gender-specific biases and barriers they may encounter during their nursing education.

The research reveals several systemic issues and institutional practices that inadvertently hinder the learning experience of men as nursing students in L&D units. Academic leaders, nurse educators, and unit managers can use these findings to review and revise policies to ensure that all nursing students are afforded equal learning opportunities during their nursing education, thereby improving the overall nursing workforce and patient care outcomes. The study can help challenge existing gender stereotypes in nursing and healthcare. By shedding light on the experiences of men in traditionally women-dominated specialties, the research contributes to breaking down societal perceptions and biases surrounding men in nursing roles.

Social role theory offers a robust explanation for the genesis of gender stereotypes and their impact on distinguishing behaviors between men and women [56]; the theory suggests that these stereotypes will only evolve when shifts in societal structures encourage individuals to embrace roles beyond traditional gender norms. Given nursing's ongoing efforts to diversify across race, ethnicity, and gender, it is crucial to pay attention to the experiences of men in the field. Prenatal, intranatal, and postnatal care for the birthing parent is essential to a healthy delivery and outcomes. In nursing, men may have much to offer in these areas, ensuring that the birthing parent has a healthy pregnancy and postnatal period caring for self and the newborn.

The current study reflects men's experiences in nursing in the U.S.; future studies should consider expanding the study beyond this geographic location. Although the sample size is small, it allows the researchers to engage with the participants and immerse themselves in the data in a way that enables them to flush out the nuances of what is said and unsaid during the interviews. The goal is not to generalize the study's findings; however, the transferability is up to the reader within their context. Withholding the racial and ethnic identification of the participants may limit the readers' connection of the findings to a specific cultural context. Therefore, future studies should consider using particular cultural groups to better reflect the cultural nuance of the participant's experiences.

## 9. Conclusion

All persons seeking to become nurses who have met all the qualifications should be able to do so without regard for outdated and gender-biased assumptions. Addressing issues related to gender and masculinity in caring professions such as nursing, especially in areas where the professional role is highly impacted by public view, requires a multifaceted approach, including education and awareness-raising efforts. Nurse educators and clinical instructors can promote greater inclusivity and diversity by creating an environment that welcomes and supports students of all genders.

This study explored men's experiences in nursing during their clinical rotations in the L&D units. The research findings provide valuable insight into the experiences of men in nursing as students, which can be used to support and improve nursing education in this area. We must note that the ability to address the emotional challenges in L&D units is not determined by gender but by the nurse's training, skills, empathy, and ability to connect with patients. Men in nursing can provide emotional support and high-quality care to patients during this vulnerable and significant time in their lives.

## Figures and Tables

**Table 1:** Interview questions.

Broad interview questions for the participants
1. Tell me about your maternal and child (L&D) clinical experience during your prelicensure nursing program.
2. In what way did your gender influence the way you were perceived or treated in the L&D unit during your clinical experience?

**Table 2:** Steps of Braun and Clarke's inductive thematic analysis.

	Steps	Description
1.	Familiarization with the data	The researchers read and re-read transcripts to become familiar with the content and identify initial ideas and patterns.
2.	Generating initial codes	Generating initial codes by identifying and labeling significant data segments related to the research question or objectives. This process entails breaking down the data into smaller parts and assigning descriptive labels to each part using specific and descriptive terminology. The researcher read the data line-by-line and noted the key concepts and descriptive terms from the data to ensure that the codes accurately reflect the content and meaning of the data.
3.	Searching for themes	The researcher identifies potential themes by reviewing the generated codes to identify patterns and connections between them and the research question.
4.	Reviewing themes	Here, the themes were reviewed for relevance, clarity, coherence, and exhaustiveness. They were then compared and contrasted, potentially merging or splitting themes to ensure clarity of ideas.
5.	Defining and naming themes	The researchers further analyze and refine the themes by reviewing and re-examining the data. They consider the themes' fit with the entire data set and assess whether any modifications are needed.
6.	Producing the report	This step involves writing up the analysis and findings clearly and coherently, using quotes and examples from the data to illustrate the themes and provide evidence for the findings.

*Note:* The content of Table 2 is adapted from Braun and Clarke [43, 44].

## Data Availability

The qualitative data supporting the findings of this study are not fully accessible due to participant confidentiality concerns. However, selected excerpts from interviews relevant to the research questions are presented within the paper. Researchers interested in accessing additional data may contact the corresponding author for consideration, subject to ethical review and adherence to participant consent agreements.

## References

[1] Harding T., North N., Perkins R. (2008). Sexualizing Men’s Touch: Male Nurses and the Use of Intimate Touch in Clinical Practice. *Research and Theory for Nursing Practice*.

[2] Zhavoronkova M., Custer B. D., Neal A., Schweitzer J., Bombardieri M. (2022). *How to Ease the Nursing Shortage in America*.

[3] Santos L. M. D. (2022). Stress, Workplace Bullying, and Career Decision of Male Nursing Students: A Qualitative Inquiry of Male Undergraduate Nursing Students. *Journal of Men’s Health*.

[4] Shudifat R., Algunmeeyn A., Shoqirat N., Alja’afreh M. (2023). The Experience of Being Male Nurse: Exploring the Enhancing Factors and Barriers of Jordanian Nursing Students. *SAGE Open Nursing*.

[5] Subu M. A., Al Yateem N., Dias J. M. (2022). Listening to the Minority: A Qualitative Study Exploring Male Students’ Perceptions of the Nursing Profession and Reasons for Choosing Nursing as a Career. *Nurse Education Today*.

[6] Taylor J., Marland G., Whitford H., Carson M., Leece R. (2022). Isolation and Marginalization: Exploring Attrition of Men in Preregistration Nursing Programs. *Journal of Nursing Education*.

[7] Whitford H. M., Marland G. R., Carson M. N. (2020). An Exploration of the Influences on Under-Representation of Male Preregistration Nursing Students. *Nurse Education Today*.

[8] National League for Nursing (2023). NLN Faculty Census Survey of Schools of Nursing Academic Year 2020-2021: Executive Summary. https://www.nln.org/docs/default-source/research-statistics/nln-faculty-census-survey-of-schools-of-nursing-academic-year-2020_2022_executive_summary8_29_2022.pdf?sfvrsn=dc89307b_3.

[9] Eagly A., Steffen V. (1984). Gender Stereotypes Stem From the Distribution of Women and Men Into Social Roles. *Journal of Personality and Social Psychology*.

[10] Kronsberg S., Bouret J. R., Brett A. L. (2017). Lived Experiences of Male Nurses: Dire Consequences for the Nursing Profession. *Journal of Nursing Education and Practice*.

[11] Evans J. (2004). Men Nurses: A Historical and Feminist Perspective. *Journal of Advanced Nursing*.

[12] Appiah S., Appiah E. O., Lamptey V. N. L. (2021). Experiences and Motivations of Male Nurses in a Tertiary Hospital in Ghana. *SAGE Open Nursing*.

[13] Evans J. A. (2002). Cautious Caregivers: Gender Stereotypes and the Sexualization of Men Nurses’ Touch. *Journal of Advanced Nursing*.

[14] Keogh B., Gleeson M. (2006). Caring for Female Patients: The Experiences of Male Nurses. *British Journal of Nursing*.

[15] O’Connor T. (2015). Men Choosing Nursing: Negotiating a Masculine Identity in a Feminine World. *The Journal of Men’s Studies*.

[16] Sasa R. I. (2019). Male Nurse: A Concept Analysis. *Nursing Forum*.

[17] Abbas S., Zakar R., Fischer F. (2020). Qualitative Study of Socio-Cultural Challenges in the Nursing Profession in Pakistan. *BMC Nursing*.

[18] Anthony A. S. (2004). Gender Bias and Discrimination in Nursing Education: Can We Change it?. *Nurse Educator*.

[19] Meadus R. J. (2000). Men in Nursing: Barriers to Recruitment. *Nursing Forum*.

[20] Weaver R., Ferguson C., Wilbourn M., Salamonson Y. (2014). Men in Nursing on Television: Exposing and Reinforcing Stereotypes. *Journal of Advanced Nursing*.

[21] Petges N., Sabio C. (2020). Perceptions of Male Students in a Baccalaureate Nursing Program: A Qualitative Study. *Nurse Education in Practice*.

[22] McLaughlin K., Muldoon O. T., Moutray M. (2010). Gender, Gender Roles and Completion of Nursing Education: A Longitudinal Study. *Nurse Education Today*.

[23] Stott A. (2007). Exploring Factors Affecting Attrition of Male Students from an Undergraduate Nursing Course: A Qualitative Study. *Nurse Education Today*.

[24] Clow K. A., Ricciardelli R., Bartfay W. J. (2014). Attitudes and Stereotypes of Male and Female Nurses: The Influence of Social Roles and Ambivalent Sexism. *Canadian Journal of Behavioural Science/Revue Canadienne des Sciences du Comportement*.

[25] Kouta C., Kaite C. P. (2011). Gender Discrimination and Nursing: Α Literature Review. *Journal of Professional Nursing*.

[26] McMillian J., Morgan S. A., Ament P. (2006). Acceptance of Male Registered Nurses by Female Registered Nurses. *Journal of Nursing Scholarship*.

[27] López-Verdugo M., Ponce-Blandón J. A., López-Narbona F. J., Romero-Castillo R., Guerra-Martín M. D. (2021). Social Image of Nursing. An Integrative Review about a yet Unknown Profession. *Nursing Reports*.

[28] Cudé G. (2004). Do Men Have a Role in Maternal-Newborn Nursing?. *AWHONN Lifelines*.

[29] Cudé G., Winfrey K. (2007). The Hidden Barrier: Gender Bias: Fact or Fiction?. *Nursing for Women’s Health*.

[30] Eswi A., El Sayed Y. (2011). The Experience of Egyptian Male Student Nurses During Attending Maternity Nursing Clinical Course. *Nurse Education in Practice*.

[31] Alshammari M., Rajagopal M., Vellolikalam C., Alshammari Z. (2023). Perception of Male Nursing Students About Their Maternity Clinical Practice: A Cross-Sectional Survey From a Nursing College. *Open Journal of Nursing*.

[32] Whiteside J., Butcher D. (2015). Not a Job for a Man: Factors in the Use of Touch by Male Nursing Staff. *British Journal of Nursing*.

[33] Englander M. (2020). Phenomenological Psychological Interviewing. *The Humanistic Psychologist*.

[34] Finlay L., Friesen N., Henriksson C., Saevi T. (2012). Debating Phenomenological Methods. *Hermeneutic Phenomenology in Education*.

[35] Matua G. A., Van Der Wal D. M. (2015). Differentiating Between Descriptive and Interpretive Phenomenological Research Approaches. *Nurse Researcher*.

[36] The Johnson & Johnson Campaign for Nursing (2016). *Discover Nursing*.

[37] Creswell J. W. (2012). *Qualitative Inquiry and Research Design: Choosing Among Five Approaches*.

[38] Patton M. Q. (2015). *Qualitative Research & Evaluation Methods: Integrating Theory and Practice*.

[39] Englander M. (2012). The Interview: Data Collection in Descriptive Phenomenological Human Scientific Research. *Journal of Phenomenological Psychology*.

[40] Malterud K., Siersma V. D., Guassora A. D. (2016). Sample Size in Qualitative Interview Studies: Guided by Information Power. *Qualitative Health Research*.

[41] Vasileiou K., Barnett J., Thorpe S., Young T. (2018). Characterising and Justifying Sample Size Sufficiency in Interview-Based Studies: Systematic Analysis of Qualitative Health Research Over a 15-year Period. *BMC Medical Research Methodology*.

[42] Creswell J. W., Creswell J. D. (2017). *Research Design: Qualitative, Quantitative, and Mixed Methods Approaches*.

[43] Braun V., Clarke V. (2006). Using Thematic Analysis in Psychology. *Qualitative Research in Psychology*.

[44] Braun V., Clarke V. (2012). Thematic Analysis. *APA Handbook of Research Methods in Psychology, Vol 2: Research Designs: Quantitative, Qualitative, Neuropsychological, and Biological*.

[45] Dwyer R., Davis I., Emerald E. (2017). *Narrative Research in Practice: Stories from the Field*.

[46] Nowell L. S., Norris J. M., White D. E., Moules N. J. (2017). Thematic Analysis: Striving to Meet the Trustworthiness Criteria. *International Journal of Qualitative Methods*.

[47] Braun V., Clarke V. (2019). Reflecting on Reflexive Thematic Analysis. *Qualitative Research in Sport, Exercise and Health*.

[48] González-García M., Lana A., Zurrón-Madera P., Valcárcel-Álvarez Y., Fernández-Feito A. (2020). Nursing Students’ Experiences of Clinical Practices in Emergency and Intensive Care Units. *International Journal of Environmental Research and Public Health*.

[49] Koenig A. M., Eagly A. H. (2014). Evidence for the Social Role Theory of Stereotype Content: Observations of Groups’ Roles Shape Stereotypes. *Journal of Personality and Social Psychology*.

[50] Juliff D., Russell K., Bulsara C. (2017). Male or Nurse, What Comes First? Challenges Men Face on Their Journey to Nurse Registration Authors. *Australian Journal of Advanced Nursing*.

[51] Powers K., Herron E. K., Sheeler C., Sain A. (2018). The Lived Experience of Being a Male Nursing Student: Implications for Student Retention and Success. *Journal of Professional Nursing*.

[52] Yang C.-I., Yu H.-Y., Chin Y.-F., Lee L.-H. (2017). There is Nothing Wrong With Being a Nurse: The Experiences of Male Nursing Students in Taiwan. *Japan Journal of Nursing Science*.

[53] Stanley D. (2012). Celluloid Devils: A Research Study of Male Nurses in Feature Films. *Journal of Advanced Nursing*.

[54] Holyoake D.-D. (2019). Similarly Different: Exploring How Male Nurses in CAMHS Experience Difference in Their Gender Performance. *Comprehensive Child and Adolescent Nursing*.

[55] Terry D., Peck B., Carden C., Perkins A. J., Smith A. (2020). Traversing the Funambulist’s Fine Line Between Nursing and Male Identity: A Systematic Review of the Factors that Influence Men as They Seek to Navigate the Nursing Profession. *European Journal of Investigation in Health, Psychology, and Education*.

[56] Croft A., Schmader T., Block K. (2015). An Underexamined Inequality: Cultural and Psychological Barriers to Men’s Engagement With Communal Roles. *Personality and Social Psychology Review*.

